# Health of Non-binary and Genderqueer People: A Systematic Review

**DOI:** 10.3389/fpsyg.2019.01453

**Published:** 2019-06-25

**Authors:** Cristiano Scandurra, Fabrizio Mezza, Nelson Mauro Maldonato, Mario Bottone, Vincenzo Bochicchio, Paolo Valerio, Roberto Vitelli

**Affiliations:** ^1^Department of Neuroscience, Reproductive Sciences and Dentistry, University of Naples Federico II, Naples, Italy; ^2^Center SInAPSi, University of Naples Federico II, Naples, Italy; ^3^Department of Humanistic Studies, University of Calabria, Cosenza, Italy

**Keywords:** non-binary, genderqueer, transgender, binary, health, stigma

## Abstract

**Background:** Non-binary and genderqueer (NBGQ) people are those who do not identify within the gender binary system (male vs. female), not falling exclusively in man/male or woman/female normative categories. A higher proportion of NBGQ people is usually found within young persons. This population is marginalized and, as such, is at risk of stigmatization and of developing negative health outcomes. As literature on the health of NBGQ people is sparse, this study aims at systematically review the limited studies on this field.

**Methods:** The research questions which guided the systematic review were: (1) What are the differences in the health levels between NBGQ and binary transgender (BT) individuals? (2) What are the differences in the health levels between NBGQ and cisgender individuals? (3) Which medical and psychological interventions are most suitable for improving NBGQ health? According to PRISMA guidelines, a systematic search was conducted in PubMed, PsycInfo, Web of Science, and Google Scholar.

**Results:** Eleven studies met the inclusion criteria for the current systematic review. Among them, 9 were focused on the health differences between NBGQ and BT individuals, 4 of the latter and 1 individually were focused on the health differences between NBGQ and cisgender individuals, and 1 was focused on the evaluation of health outcomes related to medical procedures. No studies assessed psychological interventions aimed at improving health in NBGQ individuals. All studies were cross-sectional, did not generally recruit a large sample of NBGQ individuals, and used non-probability sample design. Results related to the difference in health between NBGQ and BT were mixed; indeed, some found a better health status while others a worse one. Results related to the differences in health between NBGQ and cisgender highlighted higher health needs in NBGQ than in BT individuals. The only study analyzing the effects of medical interventions on health found that NBGQ female-assigned at birth individuals improved their quality of life after chest surgery.

**Conclusions:** Although scholars are starting to pay attention to the NBGQ health, research needs to be expanded both in terms of methodology and research contents. Clinical, health-related social policies, and research recommendations in this field are reported.

## Introduction

*Transgender* is an umbrella term referring to individuals who have a gender that differs from that normatively expected of their assigned sex [American Psychological Association (APA), 2015]. Not all transgender individuals have a binary identity, namely self-identifying as woman if they were male assigned at birth (MAAB) or man if they were female assigned at birth (FAAB) (Vitelli et al., 2017). Indeed, the term *non-binary and genderqueer* (NBGQ) refers to individuals who have a gender identity that does not fall exclusively in man/male or woman/female normative categories. NBGQ individuals identify themselves with a neither exclusively masculine nor feminine gender, and their gender identity is situated beyond the gender binary, fluctuates between genders, or rejects the gender binary (Monro, 2019).

Estimates on the prevalence of NBGQ individuals vary among studies. For instance, in a survey conducted in UK, more than a half (52%) of 14.320 transgender respondents identified themselves as NBGQ (Government Equalities Office, 2018). However, in a survey conducted in USA with a sample of 27.715 transgender individuals, more than one third (35%) of respondents identified as NBGQ (James et al., 2016). Previous studies found a generational difference, highlighting a younger age in NBGQ individuals compared with binary transgender (BT) people (James et al., 2016; Clark et al., 2018). Furthermore, NBGQ individuals tend to have a non-heterosexual sexual orientation compared with BT people (Harrison et al., 2012).

Despite literature on NBGQ population's health is growing in the last years (e.g., Vincent and Lorimer, 2018), there are still no comprehensive studies specifically addressed to such a specific segment of the general transgender population. Indeed, most of the research on the transgender health tends to consider transgender individuals as belonging to a homogenous population or to stratify them on the basis of the gender spectrum to which they identify with (trans women vs. trans men; male-to-female vs. female-to-male), thus falling within the gender binary system. Notwithstanding, Matsuno (2019) highlighted that BT identity development is different from NBGQ one. Indeed, while the BT identity development usually follows a linear path usually resulting in a transition to a male or female identity, the NBGQ identity development is more flexible and less linear as it usually does not lead to a particular and specific gender identity (Fiani, 2018). As suggested by Monro (2019), this means that NBGQ individuals are a specific population, with specific health needs and healthcare experiences.

As regards health, gender non-conformity often becomes target of oppression and stigmatization leading to negative mental and physical health outcomes (e.g., Bockting et al., 2013; Bradford et al., 2013; Scandurra et al., 2017a, 2018a). Although stigmatization is a common stressful life experience among this population (Hendricks and Testa, 2012; Scandurra et al., 2017c), transgender people exercise resilience in the face of stigma (Amodeo et al., 2015, 2018; Testa et al., 2015). Research considering stress and health in NBGQ people in comparison with BT and cisgender individuals belonging to a sexual minority identity (e.g., lesbian, gay, bisexual; LGB) are still scarce. For instance, both in UK and USA NBGQ individuals showed a lower quality of life and higher levels of current serious psychological distress than BT and cisgender individuals (James et al., 2016; Government Equalities Office, 2018). On the contrary, NBGQ individuals resulted slightly less likely to report lifetime suicide attempts than BT individuals (James et al., 2016). However, as suggested by Monro (2019), findings about NBGQ health are still inconclusive.

As regards the access to healthcare services, it is urgent to debunk a misconception about NBGQ people, or rather that they do not need to medically affirm their gender (Hansbury, 2005). On the contrary, research demonstrated that many NBGQ individuals seek hormonal or surgical treatments to feminize or masculinize their body (Beckwith et al., 2017). However, as reported by James et al. (2016), the deviation between the percentage of BT individuals who desire hormonal treatment and that of BT individuals who undergo hormonal treatment (95 vs. 71%) is smaller than that found in NBGQ individuals (49 vs. 13%). In the same vein, James et al. (2016) found that 70% of NBGQ individuals expressed the need of benefitting from a gender-related counseling, but only 31% of them had access to psychological clinical services in comparison with 73% of BT people. As suggested by Puckett et al. (2018), these differences may be due to the greater need BT people express to undergo medical interventions, but another potential reason may be that NBGQ people perceive mental and medical health professionals as unfamiliar with NBGQ identity and needs. Indeed, there is evidence that NBGQ individuals face specific challenges in the access to healthcare contexts, as they feel misunderstood by providers who often approach them from a binary concept of trans identity (Lykens et al., 2018) or experience some negative interactions characterized by misgendering and unfamiliarity with NBGQ identity and health issues (Baldwin et al., 2018). These data shed light on the difference between NBGQ and BT individuals on the healthcare access, with NBGQ people probably experiencing more and diverse barriers which still need to be explored.

As research on NBGQ health is sparse, the current study was aimed at systematically reviewing studies which empirically explored the health of NBGQ people, providing clinical and health-related social policies, identifying research gaps, and providing recommendations for future research in this area. The research questions which guided this systematic review were as follows: (1) What are the differences in the health levels between NBGQ and BT individuals? (2) What are the differences in the health levels between NBGQ and cisgender individuals? (3) Which medical and psychological interventions are most suitable for improving health in NBGQ population?

## Methods

### Search Strategy

A systematic review was performed to identify published papers on the health of NBGQ individuals using the Preferred Reporting Items for Systematic Reviews and Meta-Analyses (PRISMA) guidelines (Moher et al., 2009). Terms for NBGQ individuals (non-binary and genderqueer) were searched using the OR function and were combined with the terms related to health (health, healthcare, provider) and interventions (intervention, treatment, psychology, hormonal, and surgery) using the AND operator. Additionally, in refining our search strategy, we used also terms related to stigmatization (stigma, violence, discrimination, and abuse) as it is strictly interrelated with health outcomes (Meyer, 2007; Scandurra et al., 2017b). Furthermore, we also analyzed reference lists of significant articles to identify potential relevant papers not found with the search. The databases screened for the systematic review were PubMed, PsycINFO, and Web of Science. When the search was considered completed, some authors (NMM, MB, and VB) of the current review searched for additional articles through other sources (e.g., Google Scholar).

### Eligibility Criteria

Eligible articles included English papers published in peer-reviewed journals from 1st January 2010 to 30th June 2019, reporting data on the health of NBGQ individuals. Quantitative studies (randomized controlled trial, quasi-experimental, and observational studies), qualitative studies, mixed-method studies, and longitudinal studies were considered eligible. Reviews, meta-analyses, letters to the editor, books or book chapters, commentaries, and abstracts were excluded.

### Selection Methods

Two reviewers (CS and FM) extracted the relevant data and assessed titles and abstracts identified in the literature search. They also excluded duplicates from the dataset. Disagreements between the two reviewers were solved through the involvement of two additional reviewers (PV and RV). All studies which matched the inclusion criteria were reviewed by the authors (CS and FM) and any disagreement was settled through a discussion involving two other reviewers (PV and RV).

## Results

### Synthesis of the Studies

**Figure 1:**
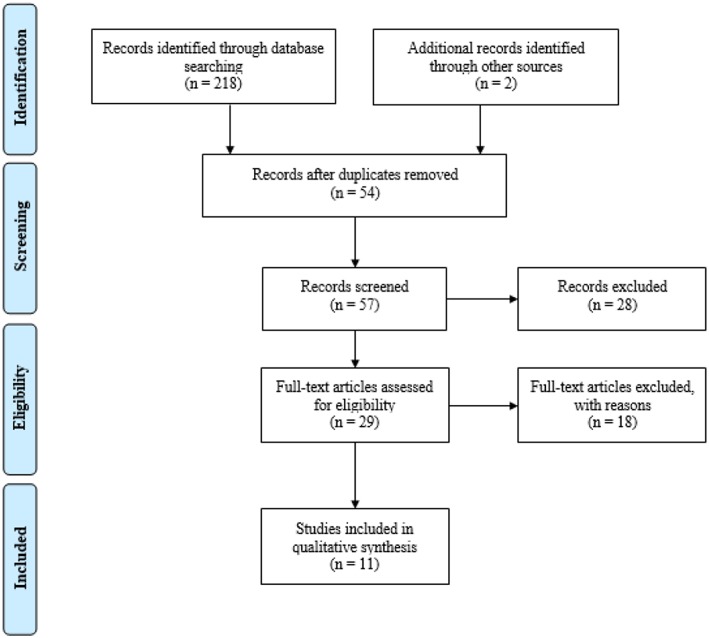
PRISMA Flowchart of the systematic search (Moher et al., 2009).

The initial search identified 218 records, as shown in the PRISMA flow diagram (Figure 1). Only 2 records were added from other sources. Among them, 54 duplicates were removed. Thus, 57 records were screened and, among them, 28 were removed for different reasons (e.g., the article was not specifically addressed to NBGQ people, authors did not differ NBGQ from BT participants, the article did not address the health outcomes of medical interventions, etc.). Among 29 full-text articles assessed for eligibility, 11 were evaluated as suitable for the current systematic review. The reasons that lead to exclude the remaining 18 papers were: (1) the research was based on a dataset already considered in another article (*n* = 1); 2) no research designs were performed (*n* = 4); (3) the article did not specifically address health outcomes (*n* = 11); (4) the journal was not peer-reviewed (*n* = 1), and (5) the identity of participants was not clearly defined (e.g., “other”) (*n* = 2).

The 11 papers included in the current systematic review are listed in Table 1. Information extracted included study design and method, main purpose, sample size, mean age of participants, country where the sample was recruited, main dimensions analyzed, and research domain. The latter is based on the research questions of the current study which considered three domains: (1) Health differences between NBGQ and BT individuals; (2) Health differences between NBGQ and cisgender individuals; and (3) Medical and psychological interventions for improving health in NBGQ individuals.

**Table 1:** Full text sources retained.

**Authors, date**	**Study design and method**	**Purpose**	**Sample size**	**Mean age**	**Country**	**Main dimensions assessed**	Research domain^*^
Agénor et al. (2018)	Quantitative; Cross-sectional online survey	Examining whether cervical cancer screening (Pap test) differs between transmasculine binary and non-binary people, both female-assigned at birth.	*N =* 122 (28 binary transmasculine and 94 non-binary transmasculine)	Non-binary transmasculine = 29.4 Binary transmasculine = 28.2 Total sample = 28.5	USA	Pap test use	1
Aparicio-García et al. (2018)	Quantitative; Cross-sectional online survey	Analyzing differences in the rate of violence, protective and health factors in non-binary, transgender, and cisgender people.	*N =* 782 (70 non-binary, 180 transgender, and 532 cisgender)	Total sample = 20.36 Differences in mean age were not reported	Spain	Violence and employment discrimination Support from family and friends Participation in activities taking place in social environments. Drug use and smoking Well-being needs Suicidal ideation	1, 2
Bradford and Catalpa (2019)	Quantitative; Cross-sectional online survey	Analyzing psychosocial distinctions between cisgender, binary transgender (trans women and men), and non-binary transgender.	*N =* 519 (153 cisgender, 102 trans women, 99 trans men, and 164 non-binary transgender)	Cisgender = 34.45 Trans women = 29.61 Trans men = 29.08 Non-binary transgender = 29.85 Total sample = 34.45	USA	Life satisfaction Gender determinism Perceived social support	1, 2
Clark et al. (2018)	Quantitative; Cross-sectional online survey	Analyzing differences in the rate of health outcomes in non-binary and binary (trans girls/women and trans boys/men) youth.	*N =* 839 (344 non-binary, 356 binary AMAB, and 139 binary AFAB)	Non-binary = 20.3 AFAB binary = 19.54 AMAB binary = 20.91	Canada	Non-suicidal self-injury Alcohol use, Marijuana use Smoking Having a family doctor, Knowledge of family doctor of trans identity Hormones use	1
Esmonde et al. (2018)	Quantitative; Retrospective review on surgery database and *post-hoc* questionnaire (cross-sectional)	Assessing postoperative health status in non-binary patients having chest affirming procedures performed.	*N =* 33 non-binary^a^	Non-binary = 29.5	USA	Quality of life Comfort with exercise Sex life Comfort in physical appearance with and without clothes	3
Goldberg et al. (2019)	Mixed; Cross-sectional online survey and focus groups	Exploring participants' mental health and health care experiences, and factors related to misgendering and less affirming treatment by providers	*N =* 506 (380 non-binary^b^ and 126 binary)	Total sample = 22.39 Differences in mean age were not reported	USA	Misgendering by providers Perceptions of providers Mental health diagnosis	1
Jones et al. (2019)	Quantitative; Cross-sectional online survey	Comparing levels of gender congruence and body satisfaction of non-binary people with binary and cisgender people.	*N =* 526 (97 nonbinary^c^, 91 binary, and 338 cisgender)	Non-binary = 32.72 Binary = 35.44 Cisgender = 36.32 Total sample = 35.70	USA	Gender congruence Body satisfaction	1, 2
Rimes et al. (2017)	Quantitative; Cross-sectional online survey	Analyzing differences in the rate of mental health and victimization in non-binary and binary young adults.	*N =* 677 (93 non-binary MAAB, 269 non-binary FAAB, 105 BT transgender females, and 210 transgender males)	Non-binary MAAB = 20.1 Non-binary FAAB = 19.9 Transgender females = 20.2 Transgender males = 19.7 Total sample = 19.9	UK	Mental health condition Self-harm Suicidality Requesting help for depression and anxiety Life satisfaction Childhood sexual abuse Domestic abuse	1
Smalley et al. (2016)	Quantitative; Cross-sectional online survey	Analyzing differences in the rate of health risk behaviors in genderqueer or non-binary, transgender women and men, and cisgender people.	*N =* 3.279 (2.954 cisgender [2.038 cisgender females; 916 cisgender males], 82 transgender women, 126 transgender men, and 117 genderqueer or non-binary).	Total sample = 29.8 Differences in mean age were not reported	USA	Diet and exercise Substance use and smoking Motor vehicle risks Sexual behaviors Violence Medical risk-taking	1, 2
Thorne et al. (2018)	Quantitative: Cross-sectional survey	Comparing levels of mental health symptomatology and social support of treatment seeking non-binary and binary individuals.	*N =* 388 (57 non-binary^d^ and 331 binary)	Non-binary = 20.02 Binary = 21.02 Total sample = 20.16	UK	Anxiety Depression Non-suicidal self-injury behavior Social support	1
Warren et al. (2016)	Quantitative: cross-sectional online survey	Analyzing differences in the rate of psychological well-being in genderqueer/non-binary and transgender females and males compared with cisgender sexual minority people.	*N =* 2.932 (2657 Cisgender, 63 transgender females, 111 transgender males, and 101 genderqueer/ non-binary).	Genderqueer/non-binary = 26.9 Transgender females = 33.32 Transgender males = 26.24 Cisgender = 29.9	USA	Accessibility, availability, and acceptability of care; Depression Anxiety Stress Perceived social support Self-esteem	2

Authors of the studies selected used different terms to refer to participants identities. The original terms were retained in the Table and the internal diversity of the subsamples was reported if indicated.

MAAB/AMAB, Male assigned at birth/Assigned male at birth; FAAB/AFAB, Female assigned at birth/Assigned female at birth.

* Research domain: 1 = Health differences between NBGQ, and BT individuals; 2 = Health differences between NBGQ and cisgender individuals; 3 = Medical and psychological intervention for improving health of NBGQ individuals.

a Participants falling under the umbrella term “non-binary” self-identified as “genderqueer,” “non-binary,” “non-conforming,” “androgynous,” or “gender fluid.”

b Participants falling under the umbrella term “nonbinary” self-identified as “nonbinary,” “genderqueer,” “gender nonconforming,” “gender fluid,” “androgynous,” “agender,” “demigender,” “third gender,” “transmasculine,” “masculine/feminine of center”, or “questioning.”

c Participants falling under the umbrella term “non-binary” self-identified as “androgynous,” “gender neutral,” “nonbinary,” “pangender,” “bigender,” “gender queer,” “gender fluid,” “intergender,” “agenderflux,” “gender creative,” or “agender.”

d Participants falling under the umbrella term “non-binary” self-identified as “Trans*,” “Gender neutral/neutrois,” “Trans,” “Non-binary gender,” “Transvestite,” “Pangender,” “Bigender,” “Genderqueer,” “Androgynous.”

Overall, studies were published between 2016 and 2019 and all of them used a cross-sectional design with quantitative methods, except for one study which supported quantitative data with focus groups. Considering the number of NBGQ individuals within the general sample recruited, the NBGQ subsample size was not generally large, as it ranged from 28 to 380 participants. However, considering the cross-sectional nature of the studies, some of them seem to have acceptable subsample size (i.e., Rimes et al., 2017; Clark et al., 2018; Goldberg et al., 2019). Looking at the NBGQ individuals' average age of all studies, participants were relatively young, as the mean age ranged from 19.9 to 32.72 years. Most of the studies were conducted in USA (*N* = 7), while 2 were conducted in UK, 1 in Spain, and 1 in Canada. Among the studies selected, 9 (81.81%) were focused on the health differences between NBGQ and BT individuals (first research domain), 4 of the latter and 1 individually (45.45%) were focused on the health differences between NBGQ and cisgender individuals (second research domain), while only 1 (9.09%) was focused on the evaluation of health outcomes related to medical procedures (i.e., cervical cancer screening and postoperative quality of life after). No studies assessed psychological interventions aimed at improving health in NBGQ individuals.

### Health Differences Between NBGQ and BT Individuals

Results related to the health difference between NBGQ and BT individuals are rather heterogeneous. Indeed, some authors generally found a better health status in NBGQ than in BT individuals (Smalley et al., 2016; Rimes et al., 2017; Agénor et al., 2018; Jones et al., 2019), while others not (Aparicio-García et al., 2018; Clark et al., 2018; Thorne et al., 2018; Bradford and Catalpa, 2019; Goldberg et al., 2019).

Specifically, considering studies which generally highlighted a better health in NBGQ individuals than BT ones, Agénor et al. (2018) found that most of their sample (77.1%) had received a Pap test in the last 3 years, with BT individuals less likely than NBGQ to have been screened to detect a uterine cancer (71.3 vs. 96.4%). As regards negative health outcomes, Rimes et al. (2017) found that NBGQ participants were less likely to have ever attempted suicide or sought help for depression and anxiety than BT participants, and that BT participants had significantly lower ratings of life satisfaction than NBGQ counterparts. In the same vein, Smalley et al. (2016) found that NBGQ individuals showed the lowest health-related risk behaviors rate (e.g., diet and exercise, avoiding medical care, etc.) compared with BT. Furthermore, Jones et al. (2019) found significantly higher levels of gender congruence and body satisfaction in NBGQ participants compared to BT counterparts.

On the contrary, as regards studies which found a worse health in NBGQ than in BT individuals, Aparicio-García et al. (2018) found that NBGQ people received lower support from family and friends than BT counterparts and that they also participated less in activities taking place in their social environments. Similarly, Bradford and Catalpa (2019) found that BT participants reported marginally higher family support scores than NBGQ participants; in addition, the correlation between friend support and life satisfaction was higher in BT participants than in NBGQ counterparts. In the study by Clark et al. (2018), several different findings were reported: (1) NBGQ participants had smaller odds (37%) of being at the higher mental health response option and a 62% increased odds of reporting non-suicidal self-injury in the past year than BT participants; (2) NBGQ individuals were more likely to report weekly alcohol use, as well as marijuana use and smoking in the past month than BT participants; (3) NBGQ participants showed smaller odds of having a family doctor, as well as a smaller odd of their family doctor knowing about their transgender identity or experience than BT counterparts; and (4) although NBGQ participants had smaller odds of reporting ever having taken hormones than BT counterparts, they had higher odds of experiencing barriers to accessing necessary hormone therapy compared to BT participants when necessary. Thorne et al. (2018) found that NBGQ individuals had higher levels of anxiety and depression, as well as lower levels of self-esteem than BT counterparts. Finally, Goldberg et al. (2019) found that NBGQ participants were more likely to report a personality and eating disorder than BT counterparts. Moreover, NBGQ participants reported more misgendering by therapists and health providers, and less trans-affirming care by health service providers compared to BT counterparts, who were 76% less likely than NBGQ participants to report being misgendered sometimes or often.

However, other inconsistent and mixed results were found. For instance, Thorne et al. (2018) did not find any significant differences neither on the likelihood of engaging in non-suicidal self-injury behavior nor on the levels of social support, thus contrasting findings obtained by Clark et al. (2018) and Aparicio-García et al. (2018), respectively. Similarly, Aparicio-García et al. (2018) did not find significant differences on drug use and smoking among groups (NBGQ vs. BT), as well as Bradford and Catalpa (2019) did not find significant differences on life satisfaction among groups, contrasting the finding obtained by Clark et al. (2018) and Rimes et al. (2017), respectively.

Finally, as regards the prejudice events, Rimes et al. (2017) found that both NBGQ FAAB and BT FAAB reported a greater rate of childhood sexual abuse than NBGQ MAAB and BT MAAB, and that NBGQ FAAB individuals reported higher rate of domestic abuse or violence than MAAB participants (both BT and NBGQ), but not than BT FAAB participants, where no difference was found. Thus, Rimes et al. (2017) highlighted a difference based more on the sex-assigned at birth than on the gender identity itself.

### Health Differences Between NBGQ and Cisgender Individuals

Results on health differences between NBGQ and cisgender individuals are a little clearer than those on the health differences between NBGQ and BT, but some contradictive findings were also reported.

Aparicio-García et al. (2018) found that, when compared with cisgender individuals, NBGQ people had a higher risk of violence and employment discrimination, were higher isolated and unhappy, had more psychological problems and higher percentage of suicidal ideation. Similarly, Smalley et al. (2016) found that NBGQ participants reported significantly higher rates of self-harm than their cisgender counterparts, and Jones et al. (2019) found that cisgender participants reported significantly higher levels of gender congruence and body satisfaction than NBGQ counterparts. In addition, Bradford and Catalpa (2019) found that cisgender participants reported higher perceived family support than NBGQ participants, and that the association between significant other and friend support and life satisfaction was higher in cisgender participants than NBGQ participants. However, the study by Warren et al. (2016) seemed to contradict these findings, as no significant differences in depression, anxiety, stress, and social support emerged between NBGQ individuals and sexual minority individuals.

Notwithstanding previous contrasting results, similarly to Aparicio-García et al. (2018), Warren et al. (2016) also found that the health needs of NBGQ individuals were higher than those perceived by cisgender counterparts, as NBGQ individuals were more likely to report a self-perceived need for mental health care, a family history of mental illness, and a personal history of mental health concerns than their cisgender counterparts (Warren et al., 2016). Thus, we can assume that NBGQ people experience more health needs than cisgender counterparts.

### Medical and Psychological Interventions for Improving the Health Status

Only one study addressing health outcomes related to medical interventions for NBGQ individuals was found. Among 458 patients undergoing gender-affirming chest surgery, Esmonde et al. (2018) found that 13% (*N* = 58) were NBGQ, all FAAB. Among them, 56% (*N* = 33) completed the post-operative questionnaire. NBGQ participants agreed that surgery improved their quality of life, comfort with exercise, sex life, and comfort in physical appearance with and without clothes. Thus, this study shows the benefits that also NBGQ individuals might experience from undergoing medical procedures. As reported before, no studies addressing specific psychological interventions (e.g., assessment, counseling, psychotherapy) for the improvement of health in NBGQ individuals were found.

## Discussion

The aim of this study was to systematically review the literature related to the health of NBGQ individuals, exploring in particular 3 domains: (1) difference in health between NBGQ and BT individuals; (2) difference in health between NBGQ and cisgender individuals; and (3) medical and psychological interventions addressed to the improvement of NBGQ health. A total of 11 studies met the inclusion criteria and were used for this systematic review.

As regards the characteristics of the selected studies, papers were recently published (from 2016), prevalently used a cross-sectional design with quantitative methods, did not generally recruited large NBGQ subsample (except for Clark et al., 2018, Goldberg et al., 2019, and Rimes et al., 2017), reported data on NBGQ individuals whose mean age ranges from 19.9 to 32.72 years, were prevalently conducted in Anglo-Saxon country (USA and UK; with the exception of two studies, one conducted in Spain and one in Canada, both in English and French), and mainly analyzed the difference in health between NBGQ and BT (i.e., first research domain). All these features seem to highlight a very recent research field which is constantly growing (Vincent and Lorimer, 2018), but still needs to be expanded, in particular in methodological designs and socio-cultural realities.

The cross-sectional nature of all selected studies represents an important bias, as it does not allow to make inferences on developmental trajectories of NBGQ individuals, as well as on causal relationships between dimensions (e.g., how stigma affects health in different stages of life and/or the role played by stable and fixed vs. unstable or not yet determined NBGQ identity). This is particularly noteworthy as it seems that NBGQ individuals analyzed in the studies selected, except for the NBGQ subsample recruited by Jones et al. (2019) whose mean age is 32.72 years, are prevalently included in a stage of life (i.e., emerging adulthood, which lasts from age 18 years to about age 29 years) that is full of developmental challenges, such as the physical and sexual maturity, the entry into university or job market, no longer being minors under the law (e.g., Arnett and Tanner, 2006; Amodeo et al., 2017; Scandurra et al., 2018b). Indeed, as suggested by Arnett et al. (2014), emerging adulthood is a period of strong instability, as youths usually experience different love relationships and frequent job changes and are far from making enduring decisions about their life. However, the flexibility and variability that characterize emerging adulthood might facilitate the exploration of sexual identity dimensions during this developmental period (Morgan, 2012), also considering that gender-related attitudes generally become more flexible in emerging adulthood than in adolescence (e.g., Davis, 2007; Marcell et al., 2011). Thus, it seems urgent to include in future studies frameworks embracing developmental perspectives (e.g., life-course perspective; Shanahan, 2000) which might allow to match the specific identity (i.e., NBGQ) with developmental challenges. In doing so, researchers should consider that LGB (e.g., Cass, 1984; D'Augelli, 1994), T (e.g., Devor, 2004; Lev, 2004), and NBGQ (e.g., Matsuno and Budge, 2017) youths have specific developmental challenges when compared with youths not belonging to a gender or sexual minority group. Specifically, NBGQ youths face unique challenges during their identity development, such as understanding how difficult may be for others to embrace a non-binary identity, feelings of invalidation and erasure of one's gender identity, managing internalized stigma, finding language categories and new narratives to describe and make meaningful their own experiences to themselves and others, and enacting a constant process of accepting their internal identity rather than being influenced by external factors (Matsuno and Budge, 2017; Bradford et al., 2018; Goldberg et al., 2019),. Furthermore, as found by Fiani and Han (2018) in a qualitative investigation on identity development, NBGQ people begin identity exploration (in terms of identity labels and self-presentation) and disclosure later than BT people, attributing this delay to a lack of information and resources regarding non-binary gender (i.e., societal awareness, role models, supportive spaces, educational materials); in addition, NBGQ people highlighted social pressure to conform and discomfort with traditional gender labeling processes more than BT people, who tended to describe more ease of identifying applicable gender norms. Thus, we can assume that NBGQ youths have to embrace a complex process to affirm themselves both internally and politically, facing with the oppression to conform to societal and normative expectations related to the gender binary system. Furthermore, studies selected are affected by a cultural bias, as they reached their samples in specific socio-cultural contexts. There are evidence that cross-cultural and regional variations in terms of social construction and expression of gender and sexuality exist, as people are always situated into specific cultural systems which, in turn, produce specific political, economic, and health inequalities (e.g., Padilla et al., 2007; Scandurra et al., 2019a). Thus, it is to be hoped that future studies will expand their samples to different geographical contexts (e.g., other EU countries beyond Spain and UK, Latin-America, Asia, and so on), as well as assume the socio-economic status as a fundamental buffering dimension.

As regards the differences in health between NBGQ and BT individuals (i.e., first research question), studies selected reported mixed findings, some finding a better health status (Smalley et al., 2016; Rimes et al., 2017; Agénor et al., 2018; Jones et al., 2019) while others a worse one (Aparicio-García et al., 2018; Clark et al., 2018; Thorne et al., 2018; Bradford and Catalpa, 2019; Goldberg et al., 2019), differently from what Matsuno and Budge (2017) reported in the only other existing review on NBGQ people. Indeed, they only reported that NBGQ individuals experience greater risk for negative mental health outcomes than their BT counterparts. However, Matsuno and Budge (2017) performed a critical and non-systematic review, writing their work before some articles included in the current review were published. Furthermore, they did not address our second (i.e., health differences between NBGQ and cisgender individuals) and third (i.e., medical and psychological intervention for improving health of NBGQ individuals) research questions, preventing us from making other comparisons. Summarizing main findings of the current review, NBGQ individuals seemed to receive more medical screenings for Pap test (Agénor et al., 2018), were less likely to attempt suicide and have higher life satisfaction (Rimes et al., 2017), as well as gender congruence and body satisfaction (Jones et al., 2019), and were less likely to engage in health-related risk behaviors (Smalley et al., 2016) than BT counterparts. As opposed to these findings, NBGQ individuals seemed to receive less support from family and friends (Aparicio-García et al., 2018; Bradford and Catalpa, 2019), and to have more negative health outcomes (non-suicidal self-injury, drug use, smoking, anxiety, depression, self-esteem, personality and eating disorders) and needs (difficulty to come out with family doctors, barriers to access hormonal therapy, and less likelihood of receiving a trans-affirming care by health service providers) (Clark et al., 2018; Thorne et al., 2018; Goldberg et al., 2019). We believe that inconsistent and mixed results in health differences between NBGQ and BT individuals might have several explanations. Indeed, all studies (1) were cross-sectional, (2) with the exception of Clark et al. (2018), Goldberg et al. (2019), and Rimes et al. (2017), did not recruited large NBGQ subsamples, (3) used non-probability sample design, and—except for Bradford and Catalpa (2019), Goldberg et al. (2019), and Smalley et al. (2016), who recruited participants even outside the lesbian, gay, bisexual, and transgender (LGBT) community (e.g., craigslist, diverse advertisements, university)—(4) recruited most of their participants within LGBT environments, preventing the possibility to access people who are not affiliated to the LGBT community. Indeed, about this last point (i.e., community-based sampling), Meyer and Wilson (2009) have argued that individuals who do not partake in the LGBT community may be different from those who do (e.g., people involved in the LGBT community may have different psychological and risk profiles than those not involved), and that the more the involvement in the LGBT community is high, the more likely one is to be sampled. All these limitations prevented the possibility to generalize results to the NBGQ population, allowing to read findings as a picture of those specific samples. While using methods that are different from cross-sectional designs (e.g., longitudinal cohort studies) and expanding the sample is quite feasible, probability sample design in gender and sexual diverse population represents a serious challenge. Indeed, as suggested by the Institute of Medicine (IOM) (2011) and Meyer and Wilson (2009), such a population is difficult to define conceptually, and many individuals are invisible as they do not come out or have reluctance to identify themselves to researchers. However, some methods exist to overcome these challenges and to improve the estimation precision of small populations, such as combining methodologically rigorous datasets or developing approximations of population patterns for findings achieved from multiple rigorous studies where non-probability samples were recruited.

Results related to the differences in health between NBGQ and cisgender individuals (i.e., second research question) are quite clearer than the previous ones. Indeed, although Warren et al. (2016) did not find significant differences related to health outcomes and social support between groups, the other four studies selected (Smalley et al., 2016; Aparicio-García et al., 2018; Bradford and Catalpa, 2019; Jones et al., 2019) found higher levels of victimization and negative health outcomes and lower levels of support in NBGQ than cisgender participants. However, the main finding is related to the higher health needs expressed by NBGQ individuals compared with cisgender counterparts. This result is in line with previous studies on transgender individuals who have been depicted as a population experiencing health disparities due to their disadvantaged social status (Reisner et al., 2014; Su et al., 2016), even if able to bounce back from adversity thanks to their resilient strategies (Singh et al., 2011, 2014; Meyer, 2015; Pflum et al., 2015). Notwithstanding, no studies analyzed whether NBGQ individuals would adopt specific individual- and community-level resilient strategies buffering the effects that stigmatizing processes have on health.

Finally, the only study analyzing the effects of medical interventions on health of NBGQ individuals (i.e., third research question) found that NBGQ FAAB individuals, contrary to the belief that they would not need to medically affirm their gender, improved their quality of life and health after chest surgery (Esmonde et al., 2018). This result is in line with previous studies which found that both hormonal treatment (e.g., Newfield et al., 2006) and chest surgery (e.g., Agarwal et al., 2018) commonly improved the quality of life and well-being of female-to-male transgender individuals. However, to the best of our knowledge, there are no studies analyzing the effects of medical interventions on NBGQ MAAB individuals. Similarly, there are no studies which empirically addressed psychological interventions specific for NBGQ individuals.

This systematic review has at least three main limitations, which should lead to read results cautiously. Indeed, as mentioned above, all studies included in the current review adopted a cross-sectional design, were performed on relatively small subsample size of NBGQ individuals, and were prevalently conducted in Anglo-Saxon countries. Such conditions prevent the opportunity to generalize results to the reference population, to establish causality among dimensions, and to expand results to different socio-cultural contexts.

### Clinical, Social, and Research Implications and Recommendations

Although results have highlighted some methodological limitations affecting the studies included in the current review, health differences between NBGQ and cisgender individuals, as well as between NBGQ and BT people, were detected. This should lead clinicians, policy makers, and researchers to view at NBGQ people as a specific population, with peculiar health needs.

From a clinical perspective, as well as for transgender individuals [e.g., American Psychological Association (APA), 2015; Edwards-Leeper et al., 2016], an affirmative practice with NBGQ people is highly recommended, especially in light of the evidence that some NBGQ individuals have access clinical services for gender-affirming treatments (Koehler et al., 2018; Taylor et al., 2018). Such a practice refers to a non-pathologizing clinical approach that accepts and validates all genders, rejecting the gender binary as a marginalizing social system, privileging some while oppressing others (Austin and Craig, 2015; Bochicchio et al., 2019; Scandurra et al., 2019b). It means that clinicians cannot presume that all transgender individuals want to live in a gender stereotypically opposed to that assigned at birth, needing medical interventions accordingly. Rather, they should deconstruct their own normative assumptions, perceiving non-binary gender identities as healthy and non-pathological, and avoiding gender binary assumptions. To our knowledge, only Nic Rider et al. (2019) published a conceptual article presenting theoretical foundations of a psychotherapy model specifically addressed to NBGQ people, called the Gender Affirmative Lifespan Approach (GALA). GALA has its theoretical roots in the health disparities theory, which asserts that therapeutically working on internalized oppression has the potentiality to improve mental health and well-being in gender diverse clients. Thus, the main aim of GALA is to promote a positive gender identity development acting through five core components: (1) building resilience; (2) developing gender literacy; (3) moving beyond the binary; (4) promoting positive sexuality; and (5), if desired, facilitating empowering connections to medical interventions. Although this psychotherapy model has the potential to become a fundamental reference in affirmative therapeutic approaches, it has not yet been empirically validated.

From a health-related social policy perspective, our findings shed light on the urgency of implementing policies to address health needs of NBGQ people, overcoming discriminatory practices and promoting health equity. First, NBGQ identities should be recognized by healthcare service systems and providers as existing and healthy identities. To this end, healthcare providers could benefit from specialized training aimed at improving the knowledge on NBGQ identities, as well as the related specific health needs (Lykens et al., 2018), thus becoming gender-literate. Furthermore, as NBGQ people are generally adolescents and young adults, healthcare providers should have an appropriate knowledge on challenges related to these specific developmental stages to provide a competent care. Second, as a concrete action, the intake forms and medical charts should be revised including not only the options for trans women and trans men, but also options such as “non-binary” or “genderqueer,” as well as giving clients the option to qualitatively describe their own gender self-identification. Similarly, such inclusive actions should be also addressed to the physical environment, making for instance restrooms gender-neutral. Third, national mental health policies should include a focus not only on transgender individuals (Veale et al., 2017), but also on NBGQ people as a population at risk, as well as developing strategies (e.g., affirmative awareness campaign) to disseminate right information and reduce health disparities.

Finally, from a research perspective, we summarize research gaps individuated, as well as recommendations for future research. We need:

1. Longitudinal cohort studies to expand our knowledge on: (a) NBGQ identity development and specific challenges, matching theoretical perspectives on gender diverse population (e.g., minority stress theory, intersectionality) with theoretical frameworks of developmental psychology (e.g., life-course perspective); (b) causal relationships between stigmatizing processes (e.g., minority stress processes), protective factors (e.g., resilience, community connectedness, activism) and health outcomes across different life stages;
2. To expand studies to geographical contexts different from USA, UK, Canada, and Spain, as well as to deepen the role of socio-economic status in health disparities in order to build culturally competent studies on NBGQ population;
3. To make every effort to recruit probability samples to allow generalization of the results to the specific population from which the sample was recruited. Although recruiting probability sample in LGBT community is a challenging task (Meyer and Wilson, 2009), an example of study that has succeeded in doing so is the Massachusetts Behavioral Risk Factor Surveillance System, a survey that was also analyzed from the transgender health perspective (e.g., Conron et al., 2012; Crissman et al., 2017);
4. To deepen both quantitatively and qualitatively the individual- and community-level resilient strategies that NBGQ individuals use to buffer the negative effects of stigma on health;
5. To understand the medical experience of NBGQ MAAB relatively to the access to surgical interventions, such as hormonal treatments or gender affirming surgeries (e.g., breast augmentation);
6. To build randomized controlled trial experiments to assess the efficacy of specific psychological interventions (e.g., psychotherapy, counseling, assessment) in improving health in NBGQ people.

## Conclusions

This systematic review shows that the scientific interest on NBGQ health is growing but, at the same time, needs to be expanded both in terms of methodology and research contents. Indeed, it was hard to obtain a clear picture of the NBGQ population in terms of health, as all selected studies used a cross-sectional design and reported data on non-probabilistic samples. Notwithstanding these limitations, studies provided valuable information on the health of NBGQ people, starting to run an innovative research field which unhooks transgender population from a gender binary system that is often reproduced in scientific research. Thus, this review may represent one of the references for future studies, which could hopefully follow the research recommendations to increase the knowledge on NBGQ health building an increasingly relevant research from both the cultural and methodological point of view, as well as informing affirmative clinical practice and social policies to reduce the health equity gap.

## Author Contributions

CS, FM, and RV conceived and designed the study and drafted the manuscript. CS and FM managed the literature searches and analyses. NM, MB, and VB refined the literature search. PV and RV solved disagreements between two main reviewers (CS and FM). All authors revised the article critically and read and approved the final manuscript.

### Conflict of Interest Statement

The authors declare that the research was conducted in the absence of any commercial or financial relationships that could be construed as a potential conflict of interest.
